# Correlation of *GLUT9* Polymorphisms With Gout Risk

**DOI:** 10.1097/MD.0000000000001742

**Published:** 2015-11-06

**Authors:** Qingxi Meng, Ji Yue, Mingfu Shang, Qunqun Shan, Jian Qi, Zhaohu Mao, Jian Li, Fan Zhang, Baolong Wang, Tingbao Zhao, Weiguo Wang

**Affiliations:** From the Department of Spinal Cord Injury, Institute of Orthopedics and Traumatology of Chinese PLA, General Hospital of Jinan Military Area Command, Jinan, Shandong, China.

## Abstract

Single nucleotide polymorphisms (SNPs) at the glucose transporter 9 (*GLUT9*) locus are clearly related to uric acid concentrations previously identified as a major cause of gout. Due to the important function of various SNPs, we hypothesized that the common *GLUT9* polymorphisms (rs16890979, rs6855911, and rs7442295) are associated with gout risk. The purpose of this investigation was to test the hypothesis.

Gout risk was estimated by calculating odds ratios and 95% confidence intervals (ORs and 95% CIs). Either the fixed- or the random-effect model was used for OR calculations. Subgroup analyses were carried out by ethnicity for rs16890979 and by gender for all SNPs.

We analyzed a total of 8 studies involving 2525 subjects for rs16890979, 2654 for rs6855911, and 2637 for rs7442295. A significantly declined risk was suggested in the meta-analyses of rs16890979 under dominant model (OR = 0.44, 95% CI = 0.34–0.58) and heterozygote model (OR = 0.44, 95% CI = 0.33–0.59). The OR was 0.41 under allele frequency model (OR = 0.41, 95% CI = 0.33–0.53). Significantly declined risk in relation to rs16890979 was also found among Asians. Similarly decreased risk was revealed for rs7442295, both in total samples and in males. However, the meta-analysis of rs6855911 revealed no significant associations.

These data seem to support the hypothesis that the risk of gout may be associated with *GLUT9* rs16890979 and rs7442295.

## INTRODUCTION

A major type of arthritis that has caught worldwide attention is gout, a most frequently diagnosed inflammatory joint disease characterized by inflammation, joint pain, chronic hyperuricemia, and painful tophi.^1–3^ An estimated 3,000,000 people above 18 years of age have been affected in the United States over the past decade.^4^ Hyperuricemia is thought of as an important risk factor for gout, a consequence of deposition of monosodium urate monohydrate crystals at the joints and adjacent tissues.^5^ A causal association of uric acid concentrations with gout has recently been identified in a sufficiently large epidemiological study.^6^ Previous research has shown that serum urate concentrations are genetically determined. According to the results of genome-wide association studies and candidate gene analyses, serum uric acid levels are markedly linked with single nucleotide polymorphisms (SNP) within the region of the glucose transporter 9 (*GLUT9*) gene.^7–9^

*GLUT9* (corresponds to *SLC2A9*) codes a protein of the GLUT9 facilitative glucose transporter family. The members of this family have a central role in maintaining glucose homeostasis. The encoded protein acts as a mediator of chondrocytes in cartilage matrices and thereby affects their survival. The human *GLUT9* could transport uric acid. Its SNPs have been identified as susceptibility factors for several diseases such as Alzheimer's disease, hyperuricemia, and gout.^10–12^ These data suggest that studies looking at sequence variations in the *GLUT9* gene may shed light on the molecular mechanisms underlying the prevalent inflammatory arthritis. However, previous conclusions on the correlation between the polymorphisms and gout risk have been called into question as a result of the inconsistency. For example, Hollis-Moffatt et al demonstrated evidence that *GLUT9* polymorphisms play a significant role in modifying the risk of gout, including rs16890979, rs11942223, rs11942223, and rs5028843.^13^ Disappointedly, this finding was not replicated among samples of Chinese ancestry.^14^ The genetic effects of *GLUT9* polymorphisms may be underestimated due to the limited sample size of published studies.

The purpose of our investigation was to clarify whether the most frequently studied SNPs (rs16890979, rs6855911, and rs7442295) are correlated with the genetic risk of gout by means of meta-analysis.

## MATERIALS AND METHODS

### Publication Search Strategy

To cover as many research articles reporting on correlation between *GLUT9* polymorphisms and gout as possible, we undertook literature searches in ISI Web of Science, Wiley Online Library, Embase, Science Direct, PubMed (Medline), and CNKI web databases, using the following combination: (glucose transporter 9 OR *GLUT9* OR *SLC2A9*) AND (polymorphism OR polymorphisms) AND (gout). We did not impose any language restrictions on the literature search. All possibly relevant studies were retrieved and their reference lists were scanned for additional articles. If the same patient population was investigated in more than 1 study, the most informative study with a larger sample was considered in further analyses.

### Inclusion and Exclusion Criteria

Selection of eligible studies was based on the predefined inclusion criteria: a case–control or cohort study addressing correlation of at least 1 *GLUT9* polymorphism of interest with gout risk; genetic data presented in the research article were sufficient to estimate the risk of gout (odds ratios and 95% confidence intervals [OR and 95% CI]). Studies were excluded if: included overlapped data with less subjects; gout risk was studied among patients only; and insufficient genetic data.

### Data Extraction

Data on first author's name, study design, country of origin, ethnicity/race, total cases and controls, count of genotypes, genotyping assays, gender distribution, source of controls, and year of publication were separately extracted by 2 of the investigators. In cases of disputes, discussion with a senior investigator was carried out to make a final decision.

### Statistical Analysis

Summary ORs and 95% CIs were estimated with an aim to examine the correlation between gout risk and *GLUT9* polymorphisms. Dominant model, allele frequency model, and heterozygote model (22 + 12 vs 11, 2 vs 1, and 12 vs 11, respectively) were tested in the meta-analysis. In order to decide if the fixed-effect model (FEM) or the random-effect model (REM) was used to estimate the pooled ORs, we detected inter-heterogeneity across the studies by using the Chi-squared-based Q-test and the I^2^ statistics.^15,16^ A *P* value < 0.05 or/and I^2^ > 50% indicated presence of heterogeneity. Under this condition, we chose the REM or calculate the pooled ORs ^17^ otherwise the FEM was selected.^18^ Subgroup analyses were carried out by ethnicity for rs16890979 and by gender for all 3 polymorphisms.

Publication bias was estimated using the funnel plots supplemented by the Egger's test, a linear regression approach to examine the funnel plot asymmetry on the natural logarithm scale of the OR.^19^ The 1-way sensitivity analysis was performed to check the robustness of meta-analysis results. Consistency with Hardy–Weinberg equilibrium (HWE) was examined among controls via the Chi-squared test.

Stata software (version 12.0, Stata Corp LP, College Station, TX) was utilized to analyze all statistical data. *P* < 0.05 was taken as the significance threshold for all tests.

## RESULTS

### Summary Description of the Eligible Studies

**FIGURE 1:**
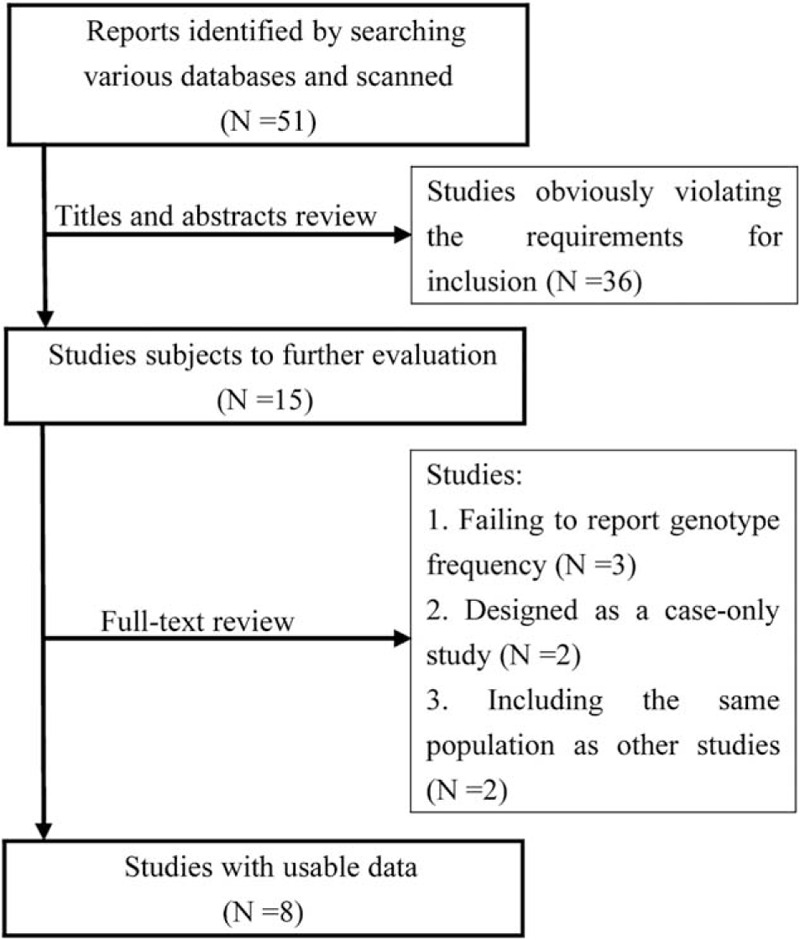
Flow chart for primary selection in this meta-analysis.

Publication searches contributed to 51 research articles. We scanned all titles and abstracts to check the eligibility, excluding 36 studies obviously not fulfilling the predescribed requirements of inclusion. Subsequent detailed evaluation resulted in another exclusion of 7 articles, because of 3 reasons: did not report genotype frequency, designed as a case-only study, and used the same cases series as other studies included in this meta-analysis. Finally, we analyzed data from 8 studies (855 cases and 1670 controls for rs16890979, 1106 cases and 1548 controls for rs6855911, and 1103 patients and 1534 control subjects for rs7442295)^13,14,20–25^ (see Fig. 1).

The distribution of genotype frequencies in the controls of all studies except for Ye et al^24^ was in agreement with HWE. Four different ethnic groups were included in the meta-analysis: Caucasian, Asian, Maori, and Pacific Islander. The studies published between 2008 and 2012 used population-based controls. In terms of the choice of genotyping assays, TaqMan was used in 4 studies (50%), PCR in 2 studies (25%), and high-resolution melting (HRM) in 2 studies (25%). In addition, most studies were carried out among male subjects (62.5%; see Table 1).

**TABLE 1:**
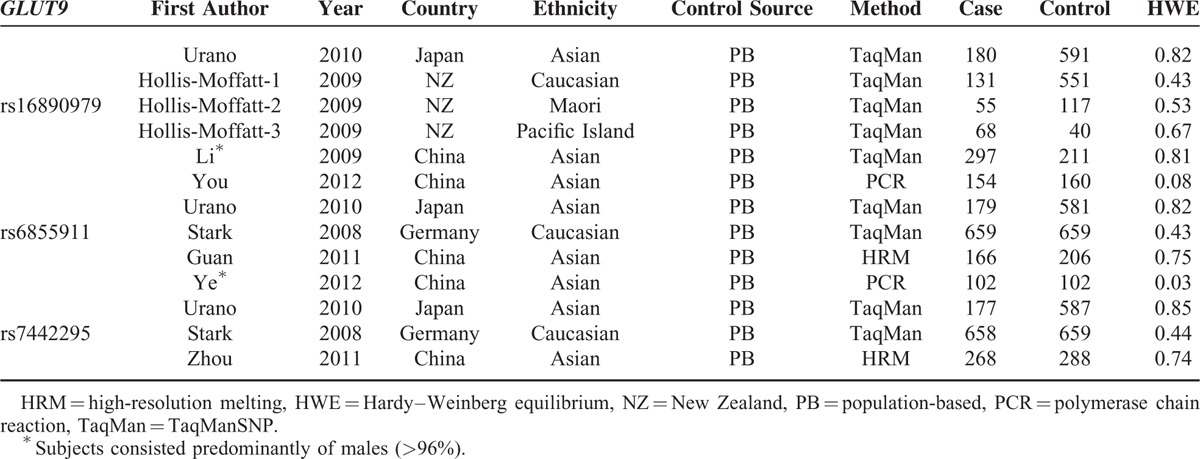
Principle Characteristics of the Studies Included in the Meta-Analysis

### Meta-Analysis Results

Results of meta-analysis examining the association between *GLUT9* polymorphisms and gout risk are shown in Table 2. As the number of rare homozygote of *GLUT9* polymorphisms in both cases and controls was zero in several studies, estimation of pooled ORs, and 95% CIs for homozygote model (22 vs 11) and recessive model (22 vs 12 + 11) was not conducted.

**TABLE 2:**
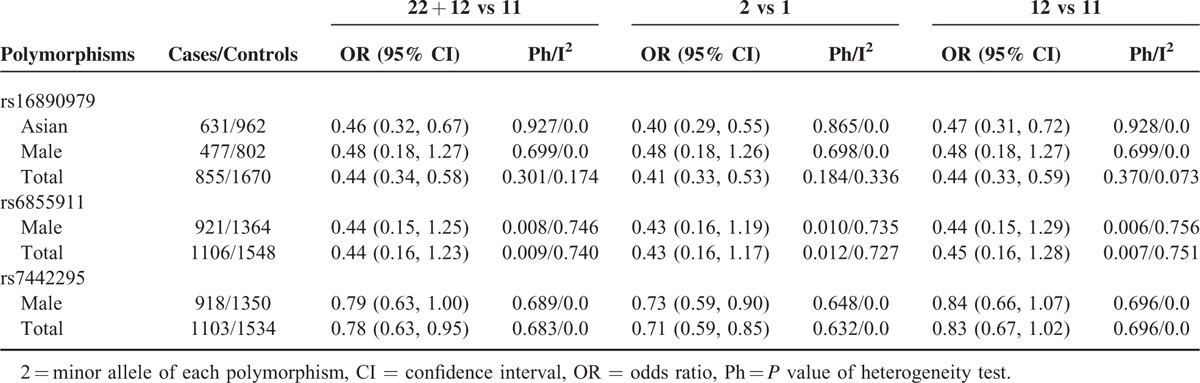
Association Between *GLUT9* Polymorphisms and Gout Risk

### Correlation Between rs16890979 and Gout Risk

**FIGURE 2:**
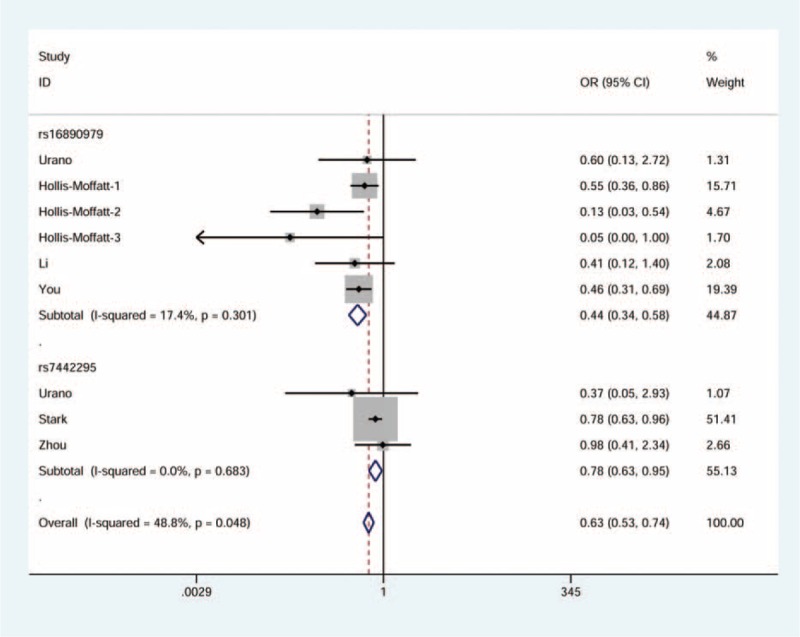
Forest plot of gout risk in association with *GLUT9* rs16890979 and rs7442295 under the dominant model. The squares and horizontal lines correspond to the study-specific odds ratio (OR) and 95% confidence interval (CI). The area of the squares reflects the weight (inverse of the variance). The diamond represents the summary OR and 95% CI.

**FIGURE 3:**
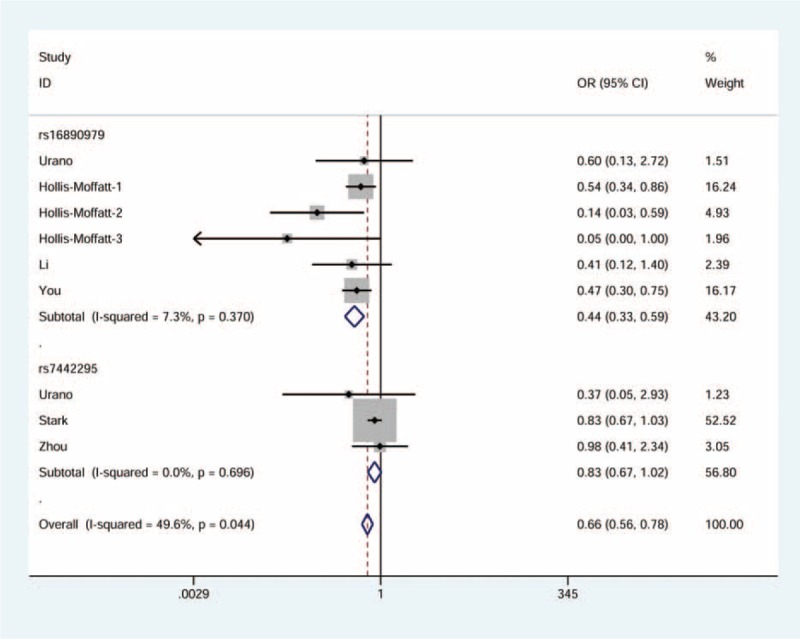
Forest plot of gout risk in association with *GLUT9* rs16890979 and rs7442295 under the heterozygous model. The squares and horizontal lines correspond to the study-specific odds ratio (OR) and 95% confidence interval (CI). The area of the squares reflects the weight (inverse of the variance). The diamond represents the summary OR and 95% CI.

**FIGURE 4:**
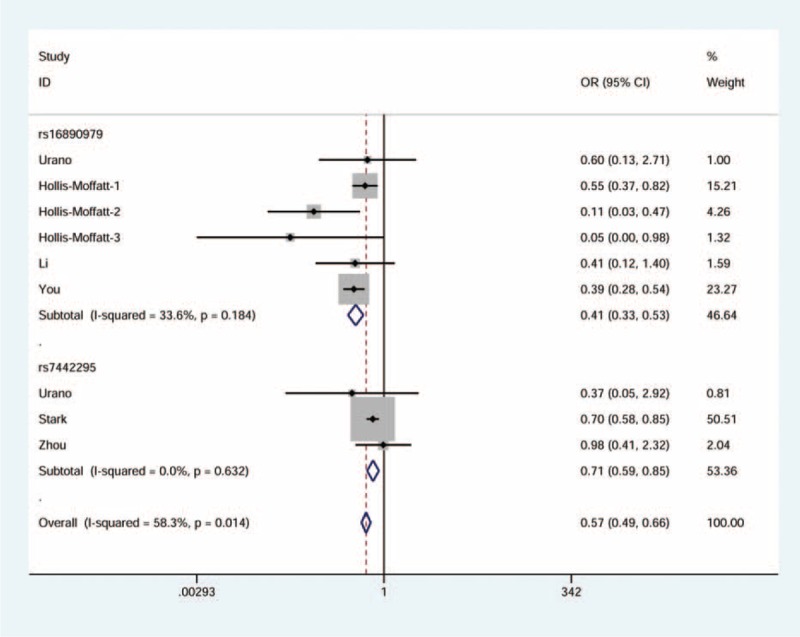
Forest plot of gout risk in association with *GLUT9* rs16890979 and rs7442295 under the allele frequency model. The squares and horizontal lines correspond to the study-specific odds ratio (OR) and 95% confidence interval (CI). The area of the squares reflects the weight (inverse of the variance). The diamond represents the summary OR and 95% CI.

There was a significant association observed between rs16890979 and the risk of gout. In general, the rare homozygote and heterozygote genotypes combined (dominant model: OR = 0.44, 95% CI = 0.34–0.58, see Fig. 2) or the heterozygote genotype alone (heterozygote model: OR = 0.44, 95% CI = 0.33–0.59, see Fig. 3) was associated with 56% declined risk of gout. Using allele frequency model, we observed a 59% decline in relation to the minor allele (OR = 0.41, 95% CI = 0.33–0.53, see Fig. 4).

Due to the available data, we performed stratified analyses by ethnicity and gender. The results showed a significant decrease in gout risk under all genetic models investigated for Asians (dominant model: OR = 0.46, 95% CI = 0.32–0.67; allele frequency model: OR = 0.40, 95% CI = 0.29–0.55; heterozygote model: OR = 0.47, 95% CI = 0.31–0.72), without substantial heterogeneity (see Table 2). By contrast, no association was indicated for males.

### Correlation Between rs6855911 and Gout Risk

The meta-analysis based on all subjects revealed a decreased risk of gout in relation to rs6855911. The decrease, however, did not reach the significance level. Likewise, no statistically significant association was indicated among males when data were stratified by gender (see Table 2).

### Correlation Between rs7442295 and Gout Risk

Figures 2–4 display the forest plots for dominant model, heterozygote model, and allele frequency model, respectively. We found a moderate decrease in the first and the third models (OR = 0.78, 95% CI = 0.63–0.95 and OR = 0.71, 95% CI = 0.59–0.85, respectively), with little heterogeneity. No statistically significant association was identified for the second model.

In the following subgroup analysis, the association remained significant only in allele frequency model for males (OR = 0.73, 95% CI = 0.59–0.90).

### Sensitivity Analyses and Publication Bias

**FIGURE 5:**
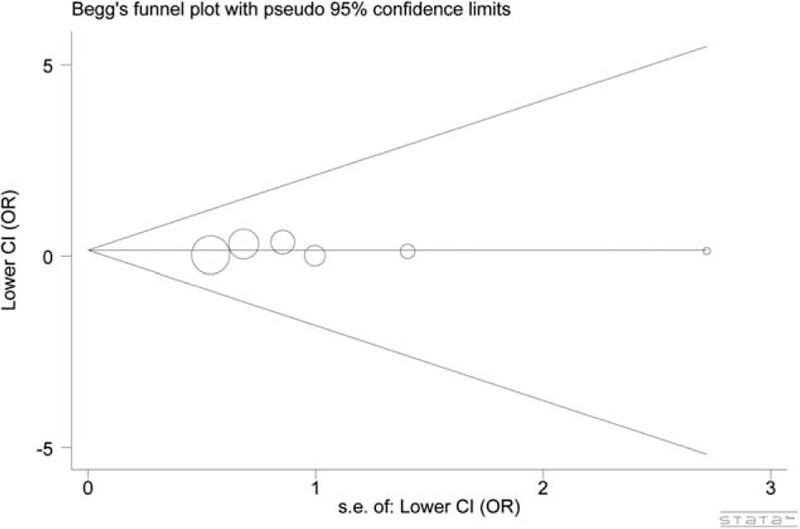
Begg plot for the assessment of potential publication bias for *GLUT9* rs16890979. There was no indication of publication bias.

The 1-way sensitivity analyses suggested that the pooled ORs were not qualitatively altered by any single study (data not shown). In the funnel plots for rs16890979 (see Fig. 5), rs6855911 (figure available on request), and rs7442295 (figure available on request), the single studies (corresponded to the circles) were symmetrically distributed. The symmetrical distribution was later confirmed by the Egger's test (*P* = 0.853, 0.360, and 0.194 for rs16890979, rs6855911, and rs7442295, respectively; dominant model). These data indicated that our meta-analysis estimates were robust and reliable.

The meta-analysis was carried out following the guidelines of PRISMA (preferred reporting items for systematic reviews and meta-analyses).^26^

## DISCUSSION

The purpose of this analysis was to investigate the hypothesis that the 3 *GLUT9* polymorphisms being investigated are associated with gout risk. On the basis of genetic and allelic data drawn from the epidemiological studies, we found a significantly declined risk of gout (OR = 0.44) associated with the rare homozygote and heterozygote genotypes combined or the heterozygote genotype alone in the meta-analysis of rs16890979, and a similar decline in relation to the minor allele (OR = 0.41). Significantly declined risk ranging from 53% to 60% was also found among Asians. Likewise, a significant decrease was revealed for rs7442295, both in total samples and in males. However, the meta-analysis of rs6855911 showed no evidence supporting a significant association. Taken together, our meta-analysis seems to support the hypothesis that rs16890979 and rs7442295, but not rs6855911 at the *GLUT9* locus, may protect against the risk to develop gout.

Aberrant expression of uric acid is a known risk factor for human diseases. Elevation in serum uric acid levels has been associated with metabolic syndrome, hypertension, cardiovascular disease, and renal disease in the domain of epidemiology.^7,27^ Based on these observations, we presumed that not only uric acid itself, but the mediators of its concentrations, such as genetic variations in candidate genes, may also play a role in these common diseases, including gout. This assumption is supported by several groups interested in family-based gout. The researchers concluded that gout arises possibly from genetic inheritance due to the high prevalence of asymptomatic hyperuricemia (range: 25–70%), a well-established predisposing factor for gout.^11,28,29^ The genetic base of gout is further evidenced in several epidemiological studies, where the investigators demonstrated strong evidence that serum levels and low renal fractional excretion of uric acid, a primary cause of hyperuricemia, are markedly linked to the polymorphisms in *GLUT9* gene.^9–11,30^ Since identifying the correlation between the nonsynonymous polymorphisms of *GLUT9* and uric acid or gout may better elucidate the mechanisms underlying gout,^31^ we decided to perform a meta-analysis to provide reliable estimates for the associations. As expected, 2 of *GLUT9* polymorphisms were significantly associated with gout risk, a finding in agreement with the data documented in previous research concerning *GLUT9* and uric acid or gout.

We additionally identified a decreased risk among males for rs7442295. This finding appears epidemiologically plausible. In a recent study, Luk and Simkin^32^ found that gout is especially prevalent among men. Lawrence et al^4^ lend further support to the fact that the inflammatory joint disease generally occurs more often among men than among women. Nevertheless, the prevalence rises with age for both, particularly among postmenopause women.^4^ Moreover, Doring et al^10^ reported that serum uric acid concentrations are differentially affected by the *GLUT9* genotypes among men and women: lower in men and higher in women. Therefore, *GLUT9* polymorphisms may predispose to gout in both genders. But it is still unclear whether the SNPs confer equal effects or the effects are more pronounced in 1 gender. Notably, gender as well as age is important component in the development of gout. It is worthwhile carrying out additional studies to determine their roles in the pathogenesis of the disease.

Due to the lack of original data for various ethnic groups, such as African and those included in the present analysis, we were unable to detect the possible associations for these ethnic populations. Even if there were some data for the Asian populations, the current number may be insufficient to provide strong evidence. This constitutes 1 limitation of our study. Second, we did not find any associations for rs6855911, which contradicts earlier observations that the polymorphisms at *GLUT9* locus are functionally important and play a role in the regulation of serum uric acid levels, a direct cause of gout. It remains to be clarified whether the null result is due to the limited sample size or the significant heterogeneity across the studies. The last but not the least, a more precise estimate could be derived if common confounding factors are taken into account, such as ethnicity, age, and gender.

In summary, to the best of our knowledge, this is the first meta-analysis examining the association of *GLUT9* polymorphisms with gout risk to date. We have demonstrated some evidence that rs16890979 and rs7442295, rather than rs6855911, may decrease the risk of developing gout. Despite the relatively reliable results, additional studies with a larger number of participants and ethnically diverse populations are recommended to determine the role of *GLUT9* gene SNPs in the pathogenesis of human gout.
